# Development and Validation of a Nomogram of In-hospital Major Adverse Cardiovascular and Cerebrovascular Events in Patients With Acute Coronary Syndrome

**DOI:** 10.3389/fcvm.2021.699023

**Published:** 2021-08-09

**Authors:** Xiangwei Bo, Yang Liu, Mingming Yang, Zhengri Lu, Yuanyuan Zhao, Lijuan Chen

**Affiliations:** ^1^Department of Cardiology, Zhongda Hospital, School of Medicine, Southeast University, Nanjing, China; ^2^School of Medicine, Southeast University, Nanjing, China; ^3^Department of Cardiology, Nanjing Lishui People's Hospital, Zhongda Hospital Lishui Branch, Southeast University, Nanjing, China

**Keywords:** ACS, nomogram, prediction model, high-risk patients, MACCE

## Abstract

**Background and Objective:** This study aims to develop and validate a nomogram for the occurrence of in-hospital major adverse cardiovascular and cerebrovascular events (MACCE) in acute coronary syndrome (ACS) patients.

**Methods:** A total of 1,360 ACS patients admitted between November 2014 and October 2019 from Zhongda Hospital and Yancheng Third People's Hospital were included. Patients admitted in Zhongda Hospital before 2018 were split into the training cohort (*n* = 793). Those admitted after 2018 in Zhongda Hospital and patients from Yancheng Third People's Hospital were split into the validation cohort (*n* = 567). Twenty eight clinical features routinely assessed including baseline characteristics, past medical history and auxiliary examinations were used to inform the models to predict in-hospital MACCE (all-cause mortality, reinfarction, stroke, and heart failure) in ACS patients. The best-performing model was tested in the validation cohort. The accuracy and clinical applicability were tested by the area under the receiver operating characteristic curve (AUC), calibration plots, and decision curve analyses (DCA).

**Results:** The in-hospital MACCE occurred in 93 (6.83%) patients. The final prediction model consists of four variables: age, Killip grading, fasting blood-glucose (FBG) and whether percutaneous coronary intervention (PCI) was performed at early stage. A nomogram was used to present the final result. Individualized nomogram exhibited comparable discrimination to the Global Registry of Acute Coronary Events (GRACE) score [AUC: 0.807 (95% CI 0.736–0.878) vs. 0.761 (95% CI 0.69–0.878)], *P* = 0.10) and a better discrimination than the Evaluation of the Methods and Management of Acute Coronary Events (EMMACE) score [AUC: 0.807 (95% CI 0.736–0.878) vs. 0.723(95% CI 0.648–0.798), *P* = 0.01] in predicting the risk of in-hospital MACCE in ACS patients. A good prediction performance was maintained in the validation cohort (AUC =0.813, 95% CI 0.738–0.889). The prediction model also exhibited decent calibration (*P* = 0.972) and clinical usefulness.

**Conclusion:** The nomogram may be a simple and effective tool in predicting the occurrence of in-hospital MACCE in ACS patients. Further longitudinal studies are warranted to validate its value in guiding clinical decision-making and optimizing the treatment of high-risk patients.

## Introduction

Coronary heart disease (CHD) is the most common cause for high mortality in the world (1), accounting for more than 40% deaths in China (2). Acute coronary syndrome (ACS) is the most severe type of CHD. The major adverse cardiovascular and cerebrovascular events (MACCE) after ACS include all-cause mortality, non-fatal myocardial infarction, non-fatal stroke, heart failure, and unplanned revascularization. Risk stratification of patients is potentially important in guiding clinical decision making and optimizing care and treatment (3–5).

There are many parameters that predict adverse outcomes after ACS, including serum biomarkers and risk scores. However, some of the serum biomarkers are not routinely tested in hospitals due to their high cost and difficulty in obtaining. In addition, sensitivity and prediction performance of these parameters are unstable in different studies (6–9). Numerous risk scores have been developed for risk stratification in patients with ACS. Commonly used risk scores include GRACE score (10), TIMI score (11) and PURSUIT score (12), etc. However, most risk scores were developed when only limited percentage of patients underwent percutaneous intervention (PCI) (13–16). With the establishment of larger number of chest pain centers across the country and current advanced medical treatments, the application of PCI has been more prevalent (17), so the risk score should be updated accordingly. Secondly, ACS has an urgent onset and the variables contained in risk scores should be available at early medical contact to all ACS patients admitted to hospital. Thirdly, many risk scores are not commonly used in clinical practice, probably because of the heavy clinical workload and the cumbersome process of using risk scores. The more streamlined the model, the better, in order to make it more user-friendly. Therefore, a contracted and updated risk score that is fitting of current clinical practice is necessitated to supplement the use of previous scoring system.

This study aimed to develop and validate a nomogram that can predict the occurrence of in-hospital MACCE in patients with ACS by using the most recent data from the Improving Care for Cardiovascular Disease in China-Acute Coronary Syndrome (CCC-ACS) project. The model incorporated the demographic, auxiliary examination, serum biomarker, past medical history, pharmacological treatment and angiographic parameters of patients. This article is written according to the TRIPOD reporting checklist (18).

## Methods

### Data Source and Participants

The baseline characteristics were registered in CCC-ACS project, a collaborative study of the American Heart Association and the Chinese Society of Cardiology launched in 2014, aimed to improve the quality of care of patients with ACS in China. Institutional review board approval was granted for this research with a waiver for informed consent by the local ethics committee. All procedures performed in this study involving human participants were in accordance with the Declaration of Helsinki.

The population included was patients admitted for ACS and registered in the CCC-ACS project. The data in Zhongda Hospital from November 2014 to October 2019 and in Yancheng Third People's Hospital from May 2015 to December 2016, which were two tertiary hospitals in China, were finally included in this study. According to the time of enrollment, patients admitted in Zhongda Hospital from November 2014 to December 2017 were serve as the training cohort (*n* = 793). Those admitted after 2018 in Zhongda Hospital and patients from Yancheng Third People's Hospital were split into the validation cohort (*n* = 567). ACS was defined as: (1) ST-segment elevation myocardial infarction (STEMI): patients with acute chest pain and persistent (>20 min) ST-segment elevation; (2) non-ST-segment elevation ACS (NSTE-ACS): patients with acute chest discomfort but no persistent ST-segment elevation exhibit ECG changes that may include transient ST-segment elevation, persistent or transient ST-segment depression, T-wave inversion, flat T waves, or pseudo-normalization of T waves; or the ECG may be normal (19, 20). Patients younger than 18 years old or with missing important variables were excluded from the analysis.

### Observation Indexes and Outcomes

Baseline clinic data, including age, gender, body mass index (BMI), clinical conditions at admission, medical history, diagnose, auxiliary examination, pre-hospital, and in-hospital treatment was derived from CCC-ACS project. From our initial list, missing data were filled by querying the hospital's electronic medical records and variables in the dataset with >30% missing values were excluded. Under the assumption that the rest of the missing data were missing-at-random, we use the multiple interpolation method for missing values. Meanwhile, we calculated the admission GRACE score. A total of 28 parameters obtained on admission were shown in Table 1. All indicators were available within 24 h of admission. The primary outcome of the study was the occurrence of MACCE, which was defined as all-cause death, reinfarction, stroke, and heart failure during hospitalization.

**Table 1:** Baseline characteristics of ACS patients with or without in-hospital MACCE.

**Characteristic**	**Training cohort**			**Validation cohort**		
	**MACCE = 0**	**MACCE = 1**	***p***	**MACCE = 0**	**MACCE = 1**	***p***
	**(*n* = 737)**	**(*n* = 56)**		**(*n* = 530)**	**(*n* = 37)**	
Male, *n* (%)	551 (74.8)	30 (53.6)	<0.01	373 (70.4)	21 (56.8)	0.08
Age, years	65 ± 13	75 ± 11.2	<0.01	64.2 ± 12.9	75.1 ± 8.5	<0.01
BMI, kg/m^2^	24.8 ± 3.1	23.7 ± 2.5	0.02	25.5 ± 3.3	24.6 ± 2.9	0.13
Clinical conditions at admission						
HR, beats/min	80 ± 16	88 ± 20	<0.01	80 ± 16	86 ± 22	0.02
SBP, mmHg	132 ± 22	130 ± 28	0.63	136 ± 25	124 ± 26	<0.01
Killip, *n* (%)			<0.01			<0.01
I	537 (72.9)	21 (37.5)		449 (84.7)	15 (40.5)	
II	143 (19.4)	13 (23.2)		64 (12.1)	12 (32.4)	
III	34 (4.6)	7 (12.5)		9 (1.7)	4 (10.8)	
IV	23 (3.1)	15 (26.8)		8 (1.5)	6 (16.2)	
Cardiogenic shock, *n* (%)	24 (3.3)	12 (21.4)	<0.01	6 (1.1)	3 (8.1)	<0.01
AHF, *n* (%)	62 (8.4)	25 (44.6)	<0.01	20 (3.8)	16 (43.2)	<0.01
Medical history, *n* (%)						
Smoke	309 (41.9)	14 (25.0)	0.01	236 (44.5)	14 (37.8)	0.43
Hypertension	478 (64.9)	43 (76.8)	0.07	317 (59.8)	21 (56.8)	0.71
COPD	12 (1.6)	4 (7.1)	<0.01	6 (1.1)	2 (5.4)	0.03
DM	226 (30.7)	24 (42.9)	0.06	122 (23.0)	11 (29.7)	0.35
History of MI	55 (7.5)	5 (8.9)	0.69	30 (5.7)	1 (2.7)	0.44
History of PCI	108 (14.7)	8 (14.3)	0.94	38 (7.2)	1 (2.7)	0.3
Renal failure	18 (2.4)	3 (5.4)	0.19	9 (1.7)	2 (5.4)	0.11
Diagnosis, *n* (%)			<0.01			<0.01
NSTEMI	277 (37.6)	33 (58.9)		144 (27.2)	9 (24.3)	
STEMI	427 (57.9)	18 (32.1)		294 (55.5)	28 (75.7)	
UAP	33 (4.5)	5 (8.9)		92 (17.4)	0 (0.0)	
Auxiliary examination						
ST-segment deviation, *n* (%)	570 (77.3)	46 (82.1)	0.41	400 (75.5)	36 (97.3)	<0.01
LVEF, %	57 ± 12	54 ± 10	0.1	57 ± 9	50 ± 10	<0.01
TnI, ng/ml	6.5 ± 15.1	6.4 ± 16.3	0.97	7.0 ± 25.6	15.0 ± 21.8	0.07
Hemoglobin, g/dl	13.6 ± 2.1	12.3 ± 2.5	<0.01	13.8 ± 1.9	12.4 ±2.4	0.27
Scr, umol/L	97 ± 84	156 ± 145	<0.01	87 ± 79	115 ± 69	0.03
FBG, mmol/L	7.0 ± 2.8	10.7 ± 6.7	<0.01	6.9 ± 2.8	7.7 ± 4.1	0.1
HDL, mmol/L	1.1 ± 0.3	1.0 ± 0.3	0.12	1.1 ± 0.3	1.2 ± 0.4	0.18
LDL, mmol/L	2.9 ± 0.9	2.7 ± 0.9	0.32	2.8 ± 1.0	2.4 ± 0.9	0.02
In-hospital treatment, *n* (%)						
PCI	574 (77.9)	25 (44.6)	<0.01	351 (66.2)	20 (54.1)	0.13
DAPT	710 (96.3)	49 (87.5)	<0.01	431 (81.3)	31 (83.8)	0.71
Pre-hospital treatment, *n* (%)						
β-blocker	60 (8.1)	3 (5.4)	0.46	31 (5.8)	4 (10.8)	0.23
ACEIs or ARBs	103 (14.0)	9 (16.1)	0.66	71 (13.4)	3 (8.1)	0.36
GRACE score	153 ± 34	192 ± 43	<0.01	139 ± 33	192 ± 38	<0.01

*Data are presented as n (%) or mean ± SD*.

*MACCE, major adverse cardiovascular and cerebrovascular events; BMI, body mass index; HR, heart rate; SBP, systolic blood pressure; AHF, acute heart failure;COPD, chronic obstructive pulmonary disease; DM, diabetes mellitus; MI, myocardial infarction; PCI, percutaneous coronary intervention; STEMI, non-ST segment elevation myocardial infarction; STEMI, ST-segment elevation myocardial infarction; UAP, unstable angina pectoris; LVEF, left ventricular ejection fraction; TnI, troponin I;Scr, serum creatinine; FBG, fasting blood-glucose; LDL, low-density lipoprotein cholesterol; DAPT, Dual antiplatelet therapy; ACEI, angiotensin converting enzyme inhibitor; ARB, angiotensin receptor inhibitor*.

### Statistical Analysis

Continuous variables were expressed as mean ± standard deviation and categorical variables were described as counts and percentages. Differences between two groups for continuous variables were compared by simple *t*-test and categorical variables were compared by exact Fisher's exact test.

Firstly, a univariate analysis was performed based on the variables available in the database to derive risk factors for the occurrence of MACCE in patients with ACS. Then, combining previously reviewed literature and clinical experience, possible risk factors were selected. A total of 28 candidate predictor variables were included to construct the prediction model. Through the Least absolute shrinkage and selection operator (LASSO) analysis, a regression penalty was applied to all variables so that the coefficients of relatively insignificant variables been excluded from the model, the independent variables that had a greater impact were selected and the corresponding regression coefficients were calculated, resulting in an optimal logistic model. The final model built to predict in-hospital MACCE in ACS patients was presented by nomogram for ease of use. The risk model was then test on the validation cohort. Model performance was assessed according to the area under the receiver operating characteristic curve (AUC), calibration plot and decision curve analyses (DCA).

All statistical tests were two-sided, and *P* < 0.05 were considered significant. Statistical analyses were completed using Stata 15.0 for Windows (StataCorp Texas, USA) and R software version3. 6. 1 (R Foundation for Statistical Computing).

## Result

### Participants

**Figure 1:**
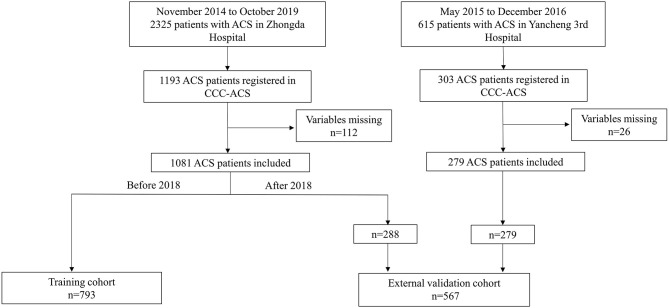
Study flow chart. Patients from Zhongda Hospital and Yancheng Third People's Hospital were included in the study. The enrolled study population was divided into a training cohort and a validation cohort. ACS, acute coronary syndrome; CCC, Improving Care for Cardiovascular Disease in China.

A total of 1,496 patients (1,193 from Zhongda Hospital and 303 from Yancheng Third People's Hospital) were consecutively registered in CCC-ACS project between November 2014 to October 2019. Patients with missing data in the variables (*n* = 138, 112 in Zhongda Hospital, 26 in Yancheng Third People's Hospital) were excluded (Figure 1), resulting in a population of 1,360 patients were included in the final analysis.

### Clinical Characteristics

Baseline characteristics of ACS Patients with or without in-hospital MACCE in the training and validation cohort are summarized in Table 1. As shown, variables significantly different between the MACCE group and non-MACCE groups were: gender, age, BMI, heart rate, Killip grading, cardiogenic shock and acute heart failure at admission, smoke, past history of chronic obstructive pulmonary disease (COPD), Hemoglobin, serum creatinine, fasting blood-glucose (FBG), early-stage percutaneous coronary intervention (PCI), and dual antiplatelet therapy (DATP) at admission. When comparing the GRACE scores, it was found that the GRACE scores in the MACCE group were significantly higher than the non-MACCE values (*p* < 0.01, Table 1).

### Outcomes

During hospitalization, 137 MACCE occurred, and a total of 93 people had MACCE, giving a MACCE incidence of 6.84%, with 40 in-hospital deaths and a mortality of 2.94%. The mean hospitalization period in training cohort was 9 ± 6 days. The incidence of MACCE did not differ between the training and validation cohort (*P* = 0.7, Table 2).

**Table 2:** Outcome of ACS Patients in training cohort and validation cohort.

**Outcome**	**Training cohort**	**Validation cohort**	***p***
	**(*n* = 793)**	**(*n* = 567)**	
Hospital stays, days	9 ± 6	9 ± 16	0.39
MACCE, *n* (%)	56 (7.1)	37 (6.5)	0.7
Mortality, *n* (%)	28 (3.5)	12 (2.1)	0.13
In-hospital HF, *n* (%)	21 (2.6)	25 (4.4)	0.08
Stroke, *n* (%)	10 (1.3)	2 (0.4)	0.08
Infarction again, *n* (%)	1 (0.1)	1 (0.2)	0.81

*Data are presented as n (%) or mean ± SD*.

*MACCE, major adverse cardiovascular and cerebrovascular events; HF, heart failure*.

### Training Model and Screening Variables

**Figure 2:**
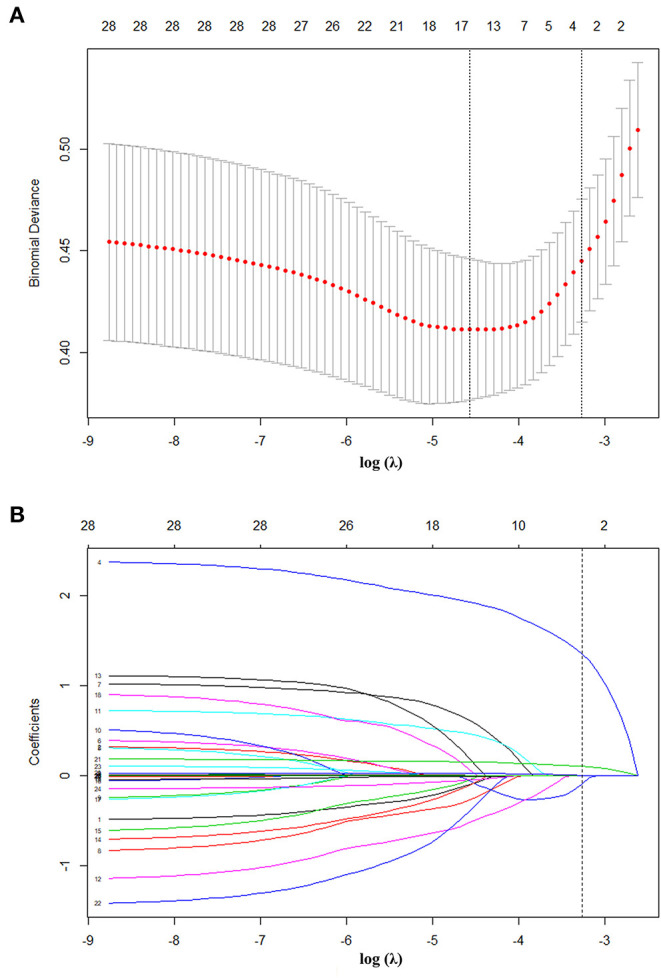
Texture feature selection using the LASSO binary logistic regression model. **(A)** Tuning parameter (λ) selection in the LASSO model used 10-fold cross-validation via minimum criteria. The AUC curve was plotted vs. log (λ). Dotted vertical lines were drawn at the optimal values by using the minimum criteria and the 1 standard error of the minimum criteria (the 1-SE criteria). A λ value of 0.038, with log (λ), 3.273 was chosen (1-SE criteria) according to 10-fold cross-validation. **(B)** LASSO coefficient profiles of the 28 texture features. A coefficient profile plot was produced against the log (λ) sequence. Vertical line was drawn at the value selected using 10-fold cross-validation, where optimal λ resulted in 4 non zero coefficients. AUC, area under the receiver operating characteristic; LASSO, least absolute shrinkage and selection operator.

**Figure 3:**
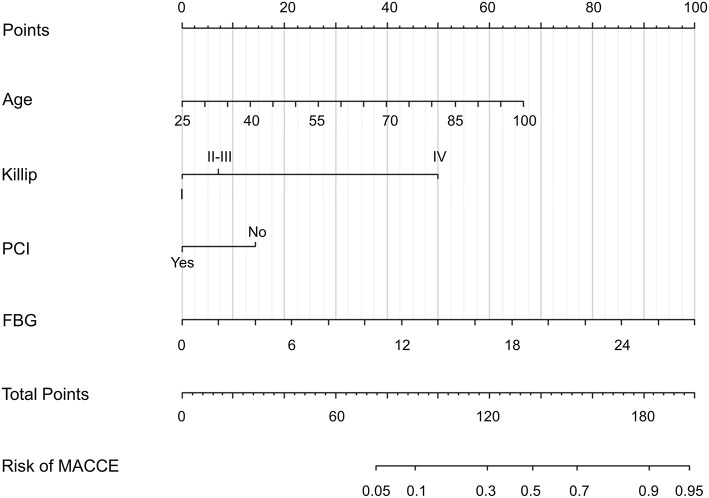
Developed in-hospital MACCE prediction nomogram in patients with ACS. ACS, acute coronary syndrome; FBG, fasting blood-glucose; MACCE, major adverse cardiovascular and cerebrovascular events; PCI, percutaneous coronary intervention.

For nomogram development, we first performed univariate analysis and a systemic literature review of existing ACS risk models, aiming to identify candidate predictor variables. Then, the available clinical and laboratory data of patients in the CCC-ACS database were reviewed. We initially selected 28 candidate variables available at 24 h of admission (Table 1). These candidate variables were reduced to four potential predictors on the basis of 793 patients in the training cohort (Figure 2). The four features were: age, Killip grading, FBG and whether PCI was performed at early medical contact and coefficients of these features in the LASSO logistic regression model were not zero. A logistic regression analysis identified the age, Killip grading, FBG and whether PCI was performed at early medical contact as independent predictors (Table 3). The model that incorporated the above predictors was presented as a nomogram (Figure 3).

**Table 3:** Logistic regression model for predicting in-hospital MACCE in ACS.

**Variable**	**β**	**Odds Ratio (95% CI)**	***P***
Age	0.346	1.413 (1.069–1.867)	0.015
Killip	0.707	2.027 (1.517–2.710)	<0.01
PCI	−0.648	0.522 (0.275–0.995)	0.048
FBG	0.161	1.174 (1.1–1.252)	<0.01
Intercept	−7.177	-	-

*Age, per 10 years; FBG, mmol/L*.

### Model Prediction Ability Evaluation

**Figure 4:**
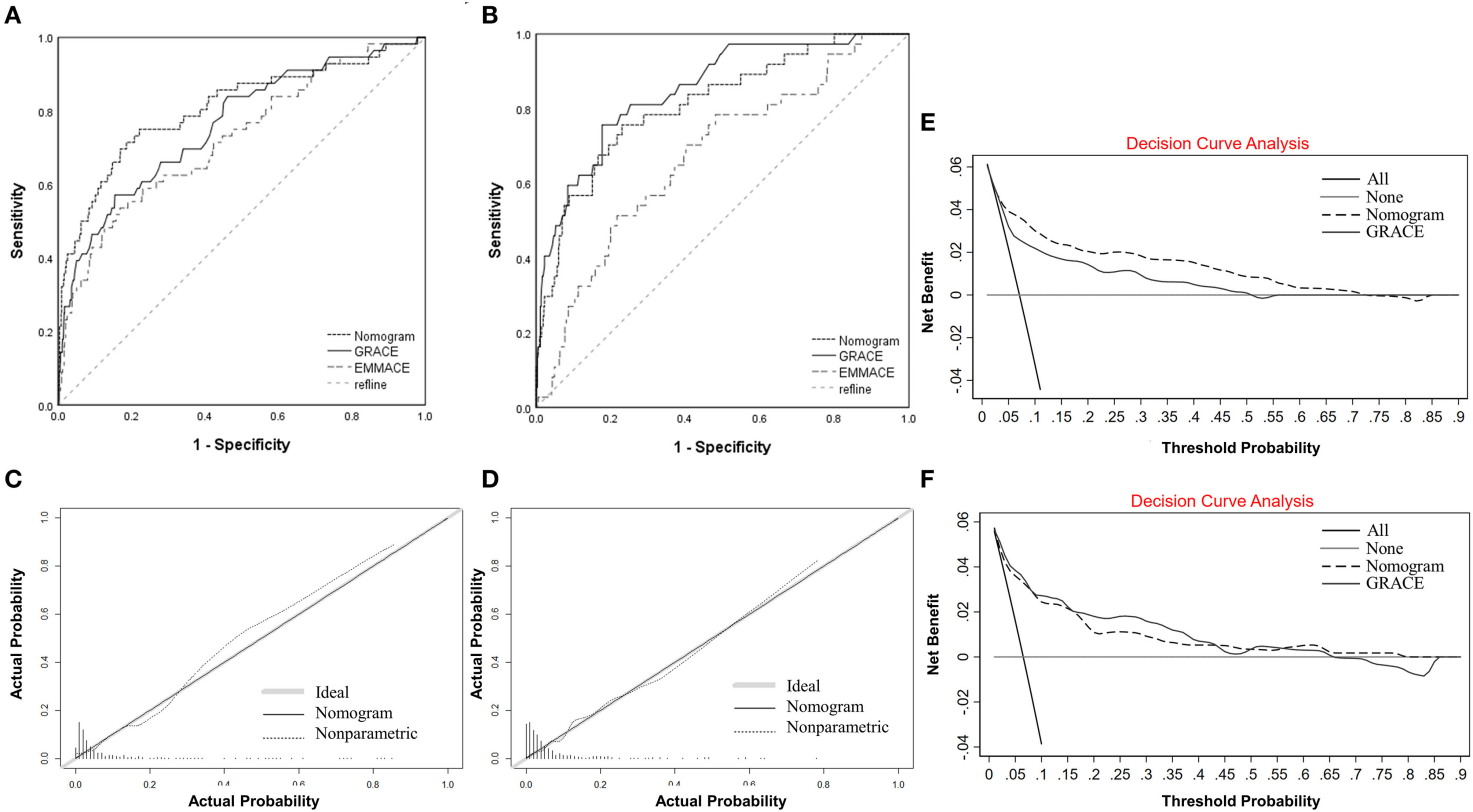
Comparison of ROC curves for nomogram with GRACE score and EMMACE score for predicting in-hospital MACCE in patients with ACS in the training cohort. **(A)** Comparison of ROC curves for the in-hospital MACCE nomogram with GRACE score and EMMACE score in the training cohort. **(B)** Comparison of ROC curves for the in-hospital MACCE nomogram with GRACE score and EMMACE score in the validation cohort. **(C)** Calibration plot of the in-hospital MACCE nomogram in the training cohort. **(D)** Calibration plot of the in-hospital MACCE nomogram prediction in the validation cohort. The x-axis represents the predicted in-hospital MACCE risk in ACS. The y-axis represents the actual diagnosed in-hospital MACCE. The diagonal line represents a perfect prediction by an ideal model. The solid line represents the performance of the nomogram, in which a closer fit to the diagonal line represents a better prediction. **(E)** Decision curve analysis for the in-hospital MACCE nomogram and GRACE score in the training cohort. **(F)** Decision curve analysis for the in-hospital MACCE nomogram and GRACE score in the validation cohort. The y-axis measures the net benefit. The black dotted line represents the in-hospital MACCE nomogram and the black zone line represents the GRACE score. The gray solid line represents the assumption that all patients are ACS complicated with MACCE. The black solid line represents the assumption that none of the patients used MACCE in ACS. The decision curve analysis showed that if the threshold probability of a patient is between 2 and 80%, using this in-hospital MACCE nomogram prediction in the current study to predict in-hospital MACCE risk adds more benefit than the “intervention-for-all” patient scheme or the “intervention-for-none”. ACS, acute coronary syndrome; EMMACE, Evaluation of the Methods and Management of Acute Coronary Events; GRACE, Global Registry of Acute Coronary Events; MACCE, major adverse cardiovascular and cerebrovascular events; ROC, receiver operating characteristic.

The AUC of the nomogram was 0.807 (95% CI 0.736–0.878, Table 4 and Figure 4A) in the training cohort, and the AUC of the GRACE score and EMMACE score were 0.761(95% CI 0.69–0.878) and 0.723 (95% CI 0.648–0.798). In the validation cohort, the nomogram yielded an AUC of 0.813 (95% CI 0.738–0.889, Table 4 and Figure 4B), and the AUC of the GRACE score and EMMACE score were 0.851(95% CI 0.786–0.916) and 0.675(95% CI 0.585–0.764), proving the discriminatory capacity of the nomogram was comparable with the GRACE score (training *P* = 0.10, validation *P* = 0.28) and superior to the EMMACE score (training *P* = 0.01, validation *P* < 0.01).

**Table 4:** The AUC (95% CI) of different models in training cohort and validation cohort.

**Cohort**		**Nomogram**	**GRACE score**	**EMMACE model**	***P*_**1**_**	***P*_**2**_**
Training cohort		0.807 (0.736–0.878)	0.761 (0.69–0.878)	0.723 (0.648–0.798)	0.10	0.01
Validation cohort	All	0.813 (0.738–0.889)	0.851 (0.786–0.916)	0.675 (0.585–0.764)	0.28	<0.01
	NSTE-ACS	0.913 (0.851–0.975)	0.852 (0.683–1.000)	0.733 (0.598–0.869)	0.41	<0.01
	STEMI	0.801 (0.712–0.890)	0.836 (0.760–0.912)	0.73(0.608–0.813)	0.33	0.03

*P_1_, Nomogram compared with GRACE score; P_2_, Nomogram compared with EMMACE model*.

Depending on whether the ST segment elevated or not, ACS patients can be divided into STEMI and NSTE-ACS cohort. The subgroup analysis showed consistent results as the overall population. The nomogram performed better in the NSTE-ACS patients than in the STEMI cohort with an AUC of 0.913 (95% CI 0.851–0.975, Table 4), higher than the AUC of 0.852 (95% CI 0.68–1.000) of the GRACE score, but the difference was not statistically significant (*P* = 0.4). In the STEMI cohort, the nomogram yielded an AUC of 0.801 (95% CI 0.712–0.890, Table 4) and the AUC of the GRACE score was 0.836(CI 0.712–0.890, 0.760–0.912), the difference was not statistically significant (*P* = 0.33). The nomogram performed better than the EMMACE score, whether validated in the ACS population or separately in subgroups, with a statistically significant difference.

Good calibration was observed for the probability of MACCE both in the training and validation cohort. The calibration plot yielded a non-significant statistic (training, *p* = 0.847 > 0.05; validation, *p* = 0.972 > 0.05, Figures 4C,D), revealed a proper consistency between nomogram predictions and actual observations.

### Clinical Application

A DCA for nomogram and GRACE score was performed to determine their clinical validity by quantifying the net benefit at different threshold probabilities in the validation cohort (Figures 4E,F).

The decision curve showed that if the threshold probability of a patient is between 2 and 80%, the net benefit of applying this nomogram is significantly higher than the two extreme cases, where all patients are considered to be at high risk or all at low risk. The net benefit of the nomogram was comparable to the GRACE score, but the nomogram has a much wider range of benefits. This model has good clinical usefulness of and of great value for evaluating the clinical prognosis of clinical patients with ACS.

## Discussion

In this study, a clinical prediction nomogram was developed and validated for predicting in-hospital MACCE in patients with ACS. The nomogram comprises four variables: age, Killip grading, FBG and whether early-stage PCI was performed during hospitalization. All indicators were available within 24 h of admission, and laboratory tests were routinely performed on patients admitted to the hospital. So that this model can predict the occurrence of in-hospital MACCE in ACS patients and provide risk stratification of patients at early medical contact without additional economic burden on patients.

Various indicators have been reported to predict prognosis of ACS: NLR (21), the hs-cTnT and hs-cTnI (22, 23), plasma ceramides (24), and progenitor cells (25), etc. Among them, some of the serological markers are not frequently tested-in hospital and demand additional time and cost. Moreover, the sensitivity and fitting degree of predicting MACCE by a single serum marker are poor and found instable in different studies. Meanwhile, several risk scores for risk assessment have been proposed, including the Evaluation of the Methods and Management of Acute Coronary Events (EMMACCE) (26), TIMI (27), PURSUIT (28), and the Global Registry of Acute Coronary Events (GRACE) risk score (29, 30). These risk scores were derived from randomized controlled trials or multinational registries and each has its own strengths. The objective of EMMACE is to evaluate care of patients admitted with acute myocardial infarction. The EMMACE risk model contains the fewest number of variables and uses three variables–age, SBP and HR on admission to predict 30-day mortality for STEMI (26). The PURSUIT risk model predicts 30-day death and the composite of death or AMI in ACS patients without persistent ST-segment elevation (28). The TIMI scores differ slightly for the different populations of NST-ACS and STEMI. The endpoint events for the NST-ACS population are composite endpoints, including mortality, myocardial infarction and emergency revascularization within 2 weeks of ACS (31). The score for STEMI patients predicts the risk of death within 30 days (11). The GRACE risk score included patients with ACS and predicts all-cause in-hospital and 6-month death (29, 30, 32). The TIMI score does not compare favorably with other scores in the prediction of NST-ACS and the prediction of prognosis in STEMI patients is comparable to that of the GRACE score (33). However, the GRACE score is more commonly used in clinical practice based on the fact that it contains more clinically relevant evaluation indicators and includes all patients with ACS (34). In these scores, the most frequently occurring indicators with the highest predictive effectiveness are age, SBP and heart rate on admission. The similarities and differences in the variables included were detailed in Supplementary Table 1.

Although these risk scores have been externally validated and some have even been recommended by guidelines for clinical practice, when applied to a “real world” population, there are still some limitations. Firstly, many risk scores are derived from randomized controlled trials that may exclude many high-risk patients or are validated only in specific populations. The mortality in this CCC-ACS cohort is higher than that in the derivation of GRACE and PURSUIT scores, which reveals the difficulties in generating “real world” data from trial populations owing to exclusions of higher-risk individuals from clinical trials. This should be considered when applying risk stratification methods to the general population (35). Secondly, the risk scores were created long ago and incorporated populations from about 20 years ago. The greater use of newer therapies for ACS has altered model discriminative performances with time. Coronary angiography and PCI were performed more widely since <30% of patients underwent PCI two decades ago when the GRACE studies conducted (10). At the same time, the application of medical treatments has changed, such as dual antiplatelet therapy, ACEIs/ARBs and β-blockers. All these changes may induce previous risk scores not being applicable to today's ACS population. Additionally, most of the patients included in the GRACE score were from Europe and The United States, among which the sample size of China or Asia was definitely limited. Whether the GRACE score is applicable in the Chinese population in the new era of widespread PCI remains to be tested. Thirdly, in clinical practice, ACS is often too urgent for risk assessment, so coronary angiography is performed directly. Moreover, many indicators included in the risk score are difficult to obtain at early medical contact. For example, testing for serum creatinine in the GRACE score takes extra time, and waiting for its results may delay the patient's condition. The high number of variables included in risk scores and the cumbersome evaluation process are also among the reasons that limit their application. Fourthly, there is currently controversy about whether risk scores can truly improve patient prognosis. A recent prospective study confirmed that the use of the GRACE risk score to guide treatment decisions in ACS increased the rate of early dumping, but does not improve the 1-year risk of death or the risk of recurrent infarction (36). This study suggests that the implementation of routine GRACE risk assessment based on guideline adherence at higher levels of cardiac centers does not benefit though may be associated with a low event rate and premature study termination. This reveals a well-recognized difficulties in generating “real world” data from trial populations and the conflict between theory and reality suggests that more exploration of risk scores is needed.

In the newly developed prediction model, age and Killip classification coincide with the evaluation indicators in the GRACE score and TIMI score (27, 29). It also emphasized on the patient's vital signs at admission, because it is closely related to the severity of the disease and the prognosis of the patients. As it is evident from the prediction model, higher FBG was risk factors for MACCE, which can also be seen from the nomogram and was the most highly co-relatable with outcomes, weighting highest among four variables included in the model. FBG is of great value in the prognosis assessment of ACS patients. Previous studies have shown that patients with hyperglycemia have a higher risk of in-hospital and long-term MACCE regardless of whether they have diabetes, which relates high FBG with the stress state and the release of inflammatory factors (37). In this study, as shown, early PCI treatment is considered to be an important protective factor for in-hospital outcomes in ACS patients. In this dataset, which is current and up-to-date, the prevalence of PCI is about 70%, which is significantly higher than 20 years ago and close to the incidence of PCI at present, demonstrating that the sample in this study represents the current population (10, 38, 39). More research is needed to explore whether early PCI can provide greater benefit to all ACS patients. The mortality rate in this study was 2.94 %, which is at a median level compared to previous studies (39, 40). The incidence of MACCE was 6.84 %, which was slightly higher than that in previous studies (39, 41), which may corelates with broader definition of MACCE in this study and patients included were from tertiary hospitals with chest pain center, indicating severe underlying conditions in patients compared to previous studies.

While developing a prediction model, the data set partition is usually necessary before its construction. Common methods include external validation from different centers, completely random grouping i.e., internal validation and external out time validation. The external validation had the highest level of evidence among all validation methods, but some of the external validation did not yield good results due to the possible genetic, environmental, and lifestyle effects of the data from different centers and the way data distributed. Dodson et al. (42) developed a prediction model for readmission within 30 days for elderly AMI patients using a completely randomized approach. Although the study included 3,006 patients from 94 hospitals in the United States, a very representative sample as a multicenter study, a completely randomized approach was used in assigning the sample. Although almost equal C-index could be obtained in the training and validation cohort statistics, it does not provide strong evidence of good predictive efficacy of this prediction model, as the completely randomized grouping may have made the characteristics of the data more similar between the two groups, reducing the variation in components. Recently, a study to predict the risk of patients with diabetic kidney disease initiating renal replacement in 3 years were grouped according to the chronological order of the data (43). In our study, a comprehensive method was used. Data from 2014 to 2017 in Zhongda Hospital was set into the training cohort, mixed data from Zhongda Hospital after 2018 years and Yancheng Third People's Hospital between 2015 and 2016 was set into the validation cohort. It was also an external validation. We did not use the data only from the Yancheng Third People's Hospital as external validation because it contained too small a sample size and an even smaller number of endpoint events, so the validation results were not representative. Also, dividing the data according to time has certain problems. As time progresses, the treatment of the disease and people's awareness of the disease may also change, which may make the two data sets differ in some characteristics. However, the comprehensive method prevented us from artificially selecting models that contribute to the test set data, making the prediction results closer to the actual clinical results. The robust and stable performance in the validation cohort of the nomogram also proved the reliability of the model.

The nomogram may serve as a complement to previous risk scores with variables that are routine available at early medical contact. Firstly, it exhibited good discrimination ability, which is comparable with the GRACE score. However, the model contains only four variables, which is easier to use than the GRACE score. Compared to the EMACCE score, the number of variables included in the model increased by only one, but the predictive power was greatly improved. Secondly, we only excluded patients with missing data, and the population included in this study was between 2014 and 2019, so the study population was the latest and closer to the real-world than other registries. This nomogram may have potential applications. It can be used to stratify patients at risk much easier and to accurately treat and care for the patient accordingly. Variables included in this model that are the same as in other models need to be highlighted in the risk stratification of patients, and predictors like FBG and early-stage PCI may provide useful information for updating other models. With the advancement of diagnosis and treatment technology, more new models are likely to emerge in the future.

## Limitation

There are some limitations of this study. Firstly, the data in this study was from a web-based data collection platform and there may be a selective bias. Although the study included data from only two centers, we did external validation in Yancheng Third People's Hospital and Zhongda Hospital has 65 group hospitals and involves patients from many parts of Anhui and Jiangsu, so the results are somewhat representative. Secondly, in the process of statistical analysis, variables with more than 30% of missing values were removed, which may lead to a certain bias in the results of statistical analysis, and to some extent, affect the objectivity and correctness of the study conclusions. For missing data, the multiple fill method was used, which to a certain extent avoided the possibility that the confidence interval range of the effect indicators might be underestimated when filling in the missing values, but the data processing techniques could only be infinitely close to the real data and could not be fully equivalent. The implementation process and data management stage of clinical trials should prevent the generation of missing data as much as possible, strengthen data collection, and avoid cases shedding missing visits, so as to ensure the integrity and validity of the data. Thirdly, the present study included a relatively modest sample size. However, the validation of the model in an internal cohort somewhat mitigates the sample size concern. In the future, more detailed studies with larger sample sizes and external verifications should be designed to further improve and confirm the accuracy of this model.

## Conclusion

Risk stratification of patients is potentially important in guiding clinical decision making and optimizing care and treatment. Here we proposed a simple and user-friendly method to objectively and accurately predict the possibility of in-hospital MACCE in ACS patients, and analysis in the validation cohort and subgroups also confirmed its forecast robustness and reliability. Although there is no evidence that it improves prognosis, more prospective observational studies are needed to confirm its clinical value.

## Data Availability Statement

The original contributions presented in the study are included in the article/Supplementary Material, further inquiries can be directed to the corresponding author.

## Ethics Statement

The studies involving human participants were reviewed and approved by Ethics Institutional Review Board of Zhongda Hospital. Written informed consent for participation was not required for this study in accordance with the national legislation and the institutional requirements. Written informed consent was not obtained from the individual(s) for the publication of any potentially identifiable images or data included in this article.

## Author Contributions

XB and LC had full access to all data used in this study and take responsibility for their integrity and the accuracy of the data analysis. XB and YL participated in data cleaning, interpreted the data, statistical analysis, developed the predictive model, prepared figures and/or tables, and wrote the first draft of the manuscript. LC and MY participated in critically revising the manuscript. ZL and YZ were responsible for filling in the clinical data of participants. All authors participated in concept and design of the present study.

## Conflict of Interest

The authors declare that the research was conducted in the absence of any commercial or financial relationships that could be construed as a potential conflict of interest.

## Publisher's Note

All claims expressed in this article are solely those of the authors and do not necessarily represent those of their affiliated organizations, or those of the publisher, the editors and the reviewers. Any product that may be evaluated in this article, or claim that may be made by its manufacturer, is not guaranteed or endorsed by the publisher.

## References

[1] Sacco RL Roth GA Reddy KS Arnett DK Bonita R Gaziano TA. The heart of 25 by 25: achieving the goal of reducing global and regional premature deaths from cardiovascular diseases and stroke: a modeling study from the American Heart Association and World Heart Federation. Glob Heart. (2016) 11:251–64. 10.1016/j.gheart.2016.04.002 27174522

[2] Zhou M Wang H Zhu J Chen W Wang L Liu S. Cause-specific mortality for 240 causes in China during 1990-2013: a systematic subnational analysis for the Global Burden of Disease Study 2013. Lancet. (2016) 387:251–72. 10.1016/S0140-6736(15)00551-6 26510778

[3] Huang Z Dong W Duan H A. probabilistic topic model for clinical risk stratification from electronic health records. J Biomed Inform. (2015) 58:28–36. 10.1016/j.jbi.2015.09.005 26370451

[4] Wilson PW D'Agostino RB Levy D Belanger AM Silbershatz H Kannel WB. Prediction of coronary heart disease using risk factor categories. Circulation. (1998) 97:1837–47. 10.1161/01.CIR.97.18.1837 9603539

[5] Tikkanen E Havulinna AS Palotie A Salomaa V Ripatti S. Genetic risk prediction and a 2-stage risk screening strategy for coronary heart disease. Arterioscler Thromb Vasc Biol. (2013) 33:2261–6. 10.1161/ATVBAHA.112.301120 23599444 PMC4210840

[6] Kahles F Ruckbeil MV Mertens RW Foldenauer AC Arrivas MC Moellmann J. Glucagon-like peptide 1 levels predict cardiovascular risk in patients with acute myocardial infarction. Eur Heart J. (2020) 41:882–9. 10.1093/eurheartj/ehz728 31620788

[7] Klingenberg R Aghlmandi S Räber L Gencer B Nanchen D Heg D. Improved risk stratification of patients with acute coronary syndromes using a combination of hsTnT, NT-proBNP and hsCRP with the GRACE score. Eur Heart J Acute Cardiovasc Care. (2018) 7:129–38. 10.1177/2048872616684678 28029055

[8] Bjørnestad E Olset H Dhar I Løland K Pedersen EKR Svingen GFT. Circulating trimethyllysine and risk of acute myocardial infarction in patients with suspected stable coronary heart disease. J Intern Med. (2020) 288:446–56. 10.1111/joim.13067 32270523

[9] Bullón P Cano-García FJ Alcocer-Gómez E Varela-López A Roman-Malo L Ruiz-Salmerón RJ. Could NLRP3-Inflammasome Be a Cardiovascular Risk Biomarker in Acute Myocardial Infarction Patients? Antioxid Redox Signal. (2017) 27:269–75. 10.1089/ars.2016.6970 27967213

[10] Steg PG Goldberg RJ Gore JM Fox KA Eagle KA Flather MD. Baseline characteristics, management practices, and in-hospital outcomes of patients hospitalized with acute coronary syndromes in the Global Registry of Acute Coronary Events (GRACE). Am J Cardiol. (2002) 90:358–63. 12161222 10.1016/s0002-9149(02)02489-x

[11] Morrow DA Antman EM Charlesworth A Cairns R Murphy SA de Lemos JA. TIMI risk score for ST-elevation myocardial infarction: A convenient, bedside, clinical score for risk assessment at presentation: An intravenous nPA for treatment of infarcting myocardium early II trial substudy. Circulation. (2000) 102:2031–7. 10.1161/01.CIR.102.17.2031 11044416

[12] Brilakis ES Wright RS Kopecky SL Mavrogiorgos NC Reeder GS Rihal CS. Association of the PURSUIT risk score with predischarge ejection fraction, angiographic severity of coronary artery disease, and mortality in a nonselected, community-based population with non-ST-elevation acute myocardial infarction. Am Heart J. (2003) 146:811–8. 10.1016/S0002-8703(03)00455-1 14597929

[13] Van de Werf F Ardissino D Betriu A Cokkinos DV Falk E Fox KA. Management of acute myocardial infarction in patients presenting with ST-segment elevation. The Task Force on the Management of Acute Myocardial Infarction of the European Society of Cardiology. Eur Heart J. (2003) 24:28–66. 10.1016/S0195-668X(02)00618-8 12559937

[14] Roffi M Patrono C Collet JP Mueller C Valgimigli M Andreotti F. 2015 ESC Guidelines for the management of acute coronary syndromes in patients presenting without persistent ST-segment elevation: Task Force for the Management of Acute Coronary Syndromes in Patients Presenting without Persistent ST-Segment Elevation of the European Society of Cardiology (ESC). Eur Heart J. (2016) 37:267–315. 10.1093/eurheartj/ehv320 26320110

[15] O'Gara PT Kushner FG Ascheim DD Casey DE Jr. Chung MK de Lemos JA. 2013 ACCF/AHA guideline for the management of ST-elevation myocardial infarction: executive summary: a report of the American College of Cardiology Foundation/American Heart Association Task Force on Practice Guidelines. Circulation (2013) 127(4):529–55. Epub 2012/12/19. 10.1161/CIR.0b013e3182742c84 23247303

[16] Amsterdam EA Wenger NK Brindis RG Casey DE Jr Ganiats TG Holmes DR Jr. 2014 AHA/ACC Guideline for the Management of Patients with Non-ST-Elevation Acute Coronary Syndromes: a report of the American College of Cardiology/American Heart Association Task Force on Practice Guidelines. J Am Coll Cardiol. (2014) 64:2713–4. 10.1161/CIR.0000000000000133 25260718

[17] Fan F Li Y Zhang Y Li J Liu J Hao Y. Chest Pain Center Accreditation Is Associated With Improved In-Hospital Outcomes of Acute Myocardial Infarction Patients in China: Findings From the CCC-ACS Project. J Am Heart Assoc. (2019) 8:e013384. 10.1161/JAHA.118.002317 31630594 PMC6898834

[18] Moons KG Altman DG Reitsma JB Ioannidis JP Macaskill P Steyerberg EW. Transparent Reporting of a multivariable prediction model for Individual Prognosis or Diagnosis (TRIPOD): explanation and elaboration. Ann Intern Med. (2015) 162:W1–73. 10.7326/M14-0698 25560730

[19] Collet JP Thiele H Barbato E Barthelemy O Bauersachs J Bhatt DL. 2020 ESC Guidelines for the management of acute coronary syndromes in patients presenting without persistent ST-segment elevation. Eur Heart J. (2020) 42:1289–367. 10.1093/eurheartj/ehaa575 32860058

[20] Ibánez B James S Agewall S Antunes MJ Bucciarelli-Ducci C Bueno H. 2017 ESC Guidelines for the management of acute myocardial infarction in patients presenting with ST-segment elevation. Revista espanola de cardiologia. (2017). 70:1082. 10.1016/j.rec.2017.11.010 29198432

[21] Zhang S Diao J Qi C Jin J Li L Gao X. Predictive value of neutrophil to lymphocyte ratio in patients with acute ST segment elevation myocardial infarction after percutaneous coronary intervention: a meta-analysis. BMC Cardiovasc Disord. (2018) 18:75. 10.1186/s12872-018-0812-6 29716535 PMC5930503

[22] Bargnoux AS Kuster N Patrier L Dupuy AM Tachon G Maurice F. Cardiovascular risk stratification in hemodialysis patients in the era of highly sensitive troponins: should we choose between hs-troponin I and hs-troponin T? Clin Chem Lab Med. (2016) 54:673–82. 10.1515/cclm-2015-0071 26457775

[23] Than MP Aldous SJ Troughton RW Pemberton CJ Richards AM Frampton CMA. Detectable high-sensitivity cardiac troponin within the population reference interval conveys high 5-year cardiovascular risk: an observational study. Clin Chem. (2018) 64:1044–53. 10.1373/clinchem.2017.285700 29760219

[24] Laaksonen R Ekroos K Sysi-Aho M Hilvo M Vihervaara T Kauhanen D. Plasma ceramides predict cardiovascular death in patients with stable coronary artery disease and acute coronary syndromes beyond LDL-cholesterol. Eur Heart J. (2016) 37:1967–76. 10.1093/eurheartj/ehw148 27125947 PMC4929378

[25] Samman Tahhan A Hammadah M Raad M Almuwaqqat Z Alkhoder A Sandesara PB. Progenitor cells and clinical outcomes in patients with acute coronary syndromes. Circ Res. (2018) 122:1565–75. 10.1161/CIRCRESAHA.118.312821 29514830 PMC5970041

[26] Dorsch MF Lawrance RA Sapsford RJ Oldham J Greenwood DC Jackson BM. A simple benchmark for evaluating quality of care of patients following acute myocardial infarction. Heart. (2001) 86:150–4. 10.1136/heart.86.2.150 11454829 PMC1729848

[27] Numasawa Y Kohsaka S Miyata H Kawamura A Noma S Suzuki M. Use of thrombolysis in myocardial infarction risk score to predict bleeding complications in patients with unstable angina and non-ST elevation myocardial infarction undergoing percutaneous coronary intervention. Cardiovasc Interv Ther. (2013) 28:242–9. 10.1007/s12928-013-0162-3 23361950

[28] Boersma E Pieper KS Steyerberg EW Wilcox RG Chang WC Lee KL. Predictors of outcome in patients with acute coronary syndromes without persistent ST-segment elevation. Results from an international trial of 9461 patients. The PURSUIT Investigators. Circulation. (2000) 101:2557–67. 10.1161/01.CIR.101.22.2557 10840005

[29] Alnasser SM Huang W Gore JM Steg PG Eagle KA Anderson FA. Jr.. Late consequences of acute coronary syndromes: global registry of acute coronary events (GRACE) Follow-up. Am J Med. (2015) 128:766–75. 10.1016/j.amjmed.2014.12.007 25554379

[30] Fox KA Fitzgerald G Puymirat E Huang W Carruthers K Simon T. Should patients with acute coronary disease be stratified for management according to their risk? Derivation, external validation and outcomes using the updated GRACE risk score. BMJ Open. (2014) 4:e004425. 10.1136/bmjopen-2013-004425 24561498 PMC3931985

[31] Antman EM Cohen M Bernink PJ McCabe CH Horacek T Papuchis G. The TIMI risk score for unstable angina/non-ST elevation MI: A method for prognostication and therapeutic decision making. Jama. (2000) 284:835–42. 10.1001/jama.284.7.835 10938172

[32] Nashef SA Roques F Michel P Gauducheau E Lemeshow S Salamon R. European system for cardiac operative risk evaluation (EuroSCORE). Eur J Cardiothorac Surg. (1999) 16:9–13. 10.1016/S1010-7940(99)00134-7 10456395

[33] D'Ascenzo F Biondi-Zoccai G Moretti C Bollati M Omedè P Sciuto F. TIMI, GRACE and alternative risk scores in Acute Coronary Syndromes: a meta-analysis of 40 derivation studies on 216,552 patients and of 42 validation studies on 31,625 patients. Contemp Clin Trials. (2012) 33:507–14. 10.1016/j.cct.2012.01.001 22265976

[34] Méndez-Eirín E Flores-Ríos X García-López F Pérez-Pérez AJ Estévez-Loureiro R Piñón-Esteban P. Comparison of the prognostic predictive value of the TIMI, PAMI, CADILLAC, and GRACE risk scores in STEACS undergoing primary or rescue PCI. Rev Esp Cardiol (Engl Ed). (2012) 65:227–33. 10.1016/j.rec.2011.10.021 22281285

[35] Wong CK White HD. Value of community-derived risk models for stratifying patients with non-ST elevation acute coronary syndromes. Eur Heart J. (2005) 26:851–2. 10.1093/eurheartj/ehi214 15781432

[36] Chew DP Hyun K Morton E Horsfall M Hillis GS Chow CK. Objective risk assessment vs standard care for acute coronary syndromes: a randomized clinical trial. JAMA Cardiol. (2021) 6:304–13. 10.1001/jamacardio.2020.6314 33295965 PMC7726696

[37] Gerbaud E Darier R Montaudon M Beauvieux MC Coffin-Boutreux C Coste P. Glycemic variability is a powerful independent predictive factor of midterm major adverse cardiac events in patients with diabetes with acute coronary syndrome. Diabetes Care. (2019) 42:674–81. 10.2337/dc18-2047 30728222

[38] Palmerini T Genereux P Caixeta A Cristea E Lansky A Mehran R. A new score for risk stratification of patients with acute coronary syndromes undergoing percutaneous coronary intervention: the ACUITY-PCI (Acute Catheterization and Urgent Intervention Triage Strategy-Percutaneous Coronary Intervention) risk score. JACC Cardiovasc Interv. (2012) 5:1108–16. 10.1016/j.jcin.2012.07.011 23174634

[39] Ran P Yang JQ Li J Li G Wang Y Qiu J. A risk score to predict in-hospital mortality in patients with acute coronary syndrome at early medicalcontact: results from the Improving Care for Cardiovascular Disease in China-Acute Coronary Syndrome (CCC-ACS) Project. Ann Transl Med. (2021) 9:167. 10.21037/atm-21-31 33569469 PMC7867931

[40] Huseynov A Baumann S Becher T Koepp J Lang S Jabbour C. Liver and cholestatic parameters as prognostic biomarkers of in-hospital MACE in patients with STEMI. Eur J Clin Invest. (2016) 46:721–9. 10.1111/eci.12655 27369447

[41] Wu Y Li S Patel A Li X Du X Wu T. Effect of a quality of care improvement initiative in patients with acute coronary syndrome in resource-constrained hospitals in china: a randomized clinical trial. JAMA Cardiol. (2019) 4:418–27. 10.1001/jamacardio.2019.0897 30994898 PMC6537808

[42] Dodson JA Hajduk AM Murphy TE Geda M Krumholz HM Tsang S. Thirty-day readmission risk model for older adults hospitalized with acute myocardial infarction. Circ Cardiovasc Qual Outcomes. (2019) 12:e005320. 10.1161/CIRCOUTCOMES.118.005320 31010300 PMC6481309

[43] Cheng Y Shang J Liu D Xiao J Zhao Z. Development and validation of a predictive model for the progression of diabetic kidney disease to kidney failure. Ren Fail. (2020) 42:550–9. 10.1080/0886022X.2020.1772294 32524865 PMC7946054

