# Supplementary material for: Development and Validation of a Nomogram of In-hospital Major Adverse Cardiovascular and Cerebrovascular Events in Patients With Acute Coronary Syndrome
Source: Front Cardiovasc Med. 2021 Aug 9;8:699023. doi: 10.3389/fcvm.2021.699023 (PMC8380764; doi:10.3389/fcvm.2021.699023)
Supplement: Supplementary file 1 [file Data_Sheet_1.PDF]

Variable	GRACE1	GRACE2	TIMI for NSTE-ACS	TIMI for STEMI	PURSUIT	CRUSADE	EMMACE	GUSTO-1	Prediction model	
**Demographic characteristics**										
Age	X	X	X	X	X		X	X	X	*
Gender					X	X				
Weight				X						
**Physical sign**										
Heart rate	X	X		X	X	X	X	X		*
Systolic blood pressure	X	X		X	X	X	X	X		*
Killip grading	X	X		X					X	*
**Auxiliary examinations**										
Abnormal ST-segment	X	X	X	X	X					*
QRS duration								X		
Cardiac injury marker	X	X	X							
Serum creatinine	X	X								
Creatinine clearance rate						X				
Hematocrit value						X				
Fasting blood-glucose									X	
**Admission status**										
Cardiac arrest	X									
Heart failure					X	X				
**Pre-admission condition**										
Severe angina			X	X						
CCS grading					X					
**Medical history**										
Previous MI		X								
Previous heart failure		X						X		
Previous AF/Arrhythmia								X		
Hypertension			X	X				X		
Diabetes mellitus			X	X		X				
High cholesterol			X							
Family history			X							
Vascular disease						X				
Previous stroke								X		
Aspirin within 1 week			X							
**Surgical factors**										
PCI		X							X	
Coronary artery stenosis≥50%			X							
**Time.**				X						
**Smoking**			X					X		
**Period**	1999-2001	1999-2005	1996-1999	1997-1998	1995-1997	2003-2006	since 1994	1990-1993	2014-2019	
**AUC**	0.64-0.95	0.66-0.85	0.59-0.88	0.62-0.87	0.67-0.81	0.71-0.88	0.77-0.78	0.754-0.8	0.73-0.94	
**Primary endpoint**	In-hospital death	6-month death	14-day MACE	30-day death	30-day death& reMI	In-hospital bleeding	30-day death	One-year survival in 30-day survivors of STEMI	In-hospital MACCE	

CCS: AF: atrial fibrillation; AUC: area under the receiver operating characteristic curve; Canadian cardiovascular society; MI: myocardial infarction; MACCE: major adverse cardiovascular and cerebrovascular events; MACE: major adverse cardiovascular events; PCI: percutaneous coronary intervention; Time: Time from symptom onset to admission.

*: variable included in≥ 4 models; X: variable included in each model.

Table I Comparison of variables included in risk scores for ACS patients
